# Relationship between the morphology of the foveal avascular zone, retinal structure, and macular circulation in patients with diabetes mellitus

**DOI:** 10.1038/s41598-018-23604-y

**Published:** 2018-03-29

**Authors:** Nathan M. Bates, Jing Tian, William E. Smiddy, Wen-Hsiang Lee, Gabor Mark Somfai, William J. Feuer, Joyce C. Shiffman, Ajay E. Kuriyan, Ninel Z. Gregori, Maja Kostic, Sandra Pineda, Delia Cabrera DeBuc

**Affiliations:** 1 Bascom Palmer Eye Institute, Miller School of Medicine, University of Miami, Miami, FL USA; 2 Retinology Unit, Pallas Kliniken, Olten, Switzerland; 3 Semmelweis University, Budapest, Hungary

## Abstract

Diabetic Retinopathy (DR) is an extremely severe and common degenerative disease. The purpose of this study was to quantify the relationship between various parameters including the Foveal Avascular Zone (FAZ) morphology, retinal layer thickness, and retinal hemodynamic properties in healthy controls and patients with diabetes mellitus (DM) with and with no mild DR (MDR) using Spectral-Domain Optical Coherence Tomography (Spectralis SDOCT, Heidelberg Engineering GmbH, Germany) and the Retinal Function Imager (Optical Imaging, Ltd., Rehovot, Israel). Our results showed a higher FAZ area and diameter in MDR patients. Blood flow analysis also showed that there is a significantly smaller venous blood flow velocity in MDR patients. Also, a significant difference in roundness was observed between DM and MDR groups supporting the development of asymmetrical FAZ expansion with worsening DR. Our results suggest a potential anisotropy in the mechanical properties of the diabetic retina with no retinopathy that may trigger the FAZ elongation in a preferred direction resulting in either thinning or thickening of intraretinal layers in the inner and outer segments of the retina as a result of autoregulation. A detailed understanding of these relationships may facilitate earlier detection of DR, allowing for preservation of vision and better clinical outcomes.

## Introduction

Diabetic retinopathy is an ocular microvascular complication of diabetes mellitus, with about one-third of diabetics being diagnosed at some point throughout their lifetime^1^. Due to a steady increase in prevalence of diabetes throughout the world, specifically in developed nations, the clinical implications of DR have resulted in it being the leading cause of vision loss in working-aged adults^2,3^. As the World Health Organization has estimated there to be 422 million diabetics worldwide; there are approximately 140 million individuals suffering from DR^4^. It is believed that one-third of DR sufferers, about 47 million inhabitants, features a severe case of the disease which is defined as vision-threatening^5^.

Nationwide, DR is a very commonly presented ocular pathology due to the predominantly high prevalence of diabetes in our society, rising due to the prevalence of obesity and increasing life span along with other factors^6^. Around 40% of people with type II diabetes and 86% of people with type I diabetes show symptoms of DR^7,8^. There is a 10-year incidence of around 75%, with 64% progressing to worse symptoms and 17% developing a severe case of retinopathy^9^.

The medical understanding of the pathophysiology and the mechanisms that promote the development of DR is constantly evolving due to research and advances in technology such as optical imaging. The development of this disorder is enhanced by numerous risk factors, including ethnic background, heredity, and hormone rich life stages including puberty and pregnancy. In general, chronic hyperglycemia, or other conditions such as hypertension, can lead to a barrage of biochemical and physiological changes, manifesting themselves through retinal dysfunction due to microvascular damage^1^. Many biochemical mechanisms have been identified as modulators of the course of DR, through which they act on cellular metabolism, signaling cascades, and growth factors^10^. Some of the implicated biochemical pathways include the accumulation of sorbitol and advanced glycation end-products (AGE), oxidative stress, protein kinase C activation, inflammation, and upregulation of the renin-angiotensin system and vascular endothelial growth factor (VEGF)^1^. The most well-understood pathway refers to VEGF, as it is considered an indisputably relevant to the pathogenesis of diabetic retinopathy.

DR is also marked by a multitude of both structural and functional retinal vasculature changes, and these changes have been easier to analyze due to advances in imaging and image processing. It has been suggested that there is an enlargement of the FAZ and a decrease in capillary blood flow velocity in patients with proliferative DR^11^. It has also been reported that these changes manifest themselves in the thickness of the retinal layers, with thinning being present for selected layers^12–20^.

The fovea is a highly-specialized region of the macula that is the central point of visual acuity. It is comprised mainly of photoreceptors with an area that features no capillary networks – this region is called the foveal avascular zone^21^. The FAZ is surrounded by interconnected capillary networks, allowing for visual detection of the region due to the contrast between the vascular-free and vascular-rich zones^12^. The noninvasive visualization of the fovea capillary network with advanced optical imaging has facilitated a better understanding of the interdependence between the FAZ and the foveal pit morphology in different systemic diseases like diabetes mellitus^22–28^. Also, the development of the FAZ and retinal structure in different weeks of gestation were studied by Hammer *et al*.^29^. A smaller FAZ and narrower foveal pit with one layer of interconnected capillaries was found in preemies with retinopathy of prematurity when compared with full term babies^30^. A higher variability between the FAZ and retinal thickness measures was also reported in healthy subjects^12,23^. Although many studies have demonstrated that there is a variety of changes in FAZ parameters between patients, including shape and area; there are no studies that have investigated the relationship between the FAZ parameters, retinal foveal morphology, and hemodynamic parameters^31–35^. The FAZ is extremely sensitive to biological events and tracking the morphology of this region can provide insights into possible pathologic processes, specifically FAZ enlargement in DR^20^. Therefore, exploring the relationship between the FAZ characteristics and onset and progression of disease may offer an early diagnostic tool that might allow for early detection before irreversible changes occur.

Optical Coherence Tomography (OCT) is one of the most commonly utilized diagnostic tools in the practice of ophthalmology, providing a non-invasive and high-resolution approach to serve as a diagnostic technique for a variety of retinal pathologies^36^. This utility has been expanded into the realm of research, facilitating researcher’s ability to track the course of the disease and better assess the *in-vivo* conditions of their study patients. OCT is an optical imaging modality that is analogous to ultrasound, however using light instead of acoustics. It deduces images based on measuring the backscattered and back-reflected light following a pulse of light. OCT can image the anterior of the eye and retina, with specific interest in the fovea and optic disk^37,38^. This modality allows for the imaging of the retinal layers, with subtle boundaries able to be detected and studied individually^39–41^. Over the past few years, OCT technology has been greatly improved regarding speed, resolution, and sensitivity, allowing for retinal structures to be viewed within seconds to a resolution of 2 microns.

Visualizing and quantizing the function and morphology of the retinal capillary network can be an extremely useful tool in the early diagnosis of a variety of pathologies, including DR^42,43^. Many techniques have been developed over time to acquire higher quality images to aid in this early detection, with the current gold standard being Fluorescein Angiography (FA)^44^. Improvements in the analysis of images obtained with FA technology has facilitated the automated leakage pattern analysis using a framework that can automatically detect various types of leakage in areas of microaneurysms or even in more advanced stages of DR^45^. However, this modality is limited in the fact that it is used repeatedly throughout treatment, which results in a higher risk of complications related to the fluorescein dye used in the procedure^46^. In contrast, the Retinal Function Imager (RFI, Optical Imaging, Ltd., Rehovot, Israel) utilizes a noninvasive method of imaging through acquiring images at a wavelength that is absorbed strongly by the hemoglobin that is found in the red blood cells (RBCs)^47^. The system is composed of a standard fundus camera that is bolstered by a stroboscopic flash lamp along with a digital camera with the ability to rapidly acquire images. By capturing reflectance changes as a function of time, the system can track the motion of RBCs and use this information to map capillaries in a resulting perfusion map visually^48^.

This study investigated the relationship between changes in both the retinal structure and circulation as well as the foveal morphology associated with diabetes. FAZ dimension measurements (e.g., area and diameter) as well as the foveal pit profile features such as central foveal thickness, foveal photoreceptor thickness, and the maximum retinal thickness were measured using the OCT device (Spectralis SDOCT, Heidelberg Engineering GmbH, Germany). Also, hemodynamic measurements such as blood flow velocity of the retinal vessels and capillary network details were measured using the RFI system.

## Results

### FAZ Dimensions

**Figure 1:**
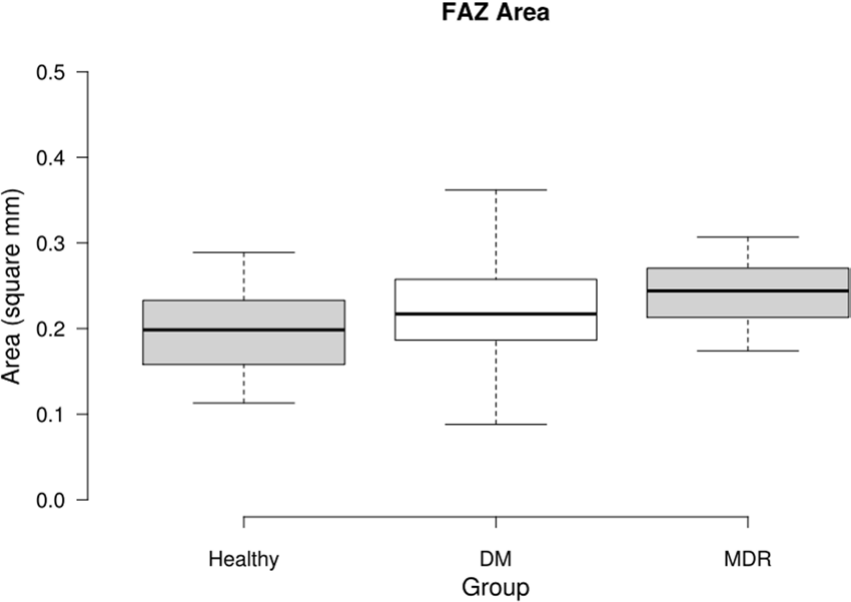
Box Plot displaying the FAZ area data for the three study groups. The middle 50% of the data groups are as follows: Healthy (0.1585–0.2398), DM (0.1770–0.2575), and MDR (0.2300–0.2795).

Various parameters regarding the FAZ region were determined using ImageJ. A summary of the results for the three study groups is presented in Table 1 (first three columns), with the mean value (±SD) being presented. ANOVA showed significance in all parameters. The results were analyzed comparing all three study groups using a Kruskal-Wallis test. The SD method was used to detect outliers in the data (mean ± 3 SD). Table 1 (last three columns) shows the p-values acquired in this analysis. The significance was set at p = 0.05. Significant results are bolded. Figure 1 shows the FAZ area’s box plot trend for the three study groups analyzed, revealing a larger FAZ when retinopathy is an eye complication in subjects with DM.

**Table 1:** FAZ Dimension Data for all groups (first three columns) and Kruskal-Wallis Results for FAZ Data (last three columns). P-values of less than 0.0001, the statistical limit of our testing, are represented as <0.0001.

Descriptor	Healthy	DM	MDR	Healthy vs. DM*	Healthy vs. MDR*	DM vs. MDR*
Area (mm^2^)	0.196 ± 0.052	0.218 ± 0.063	0.257 ± 0.051	0.22	0.0004 (<)	0.0025 (<)
Perimeter (mm)	2.02 ± 0.247	2.17 ± 0.373	2.52 ± 0.381	0.13	^**‡**^0.0001 (<)	^**‡**^0.0001 (<)
Circumference (mm)	0.605 ± 0.127	0.585 ± 0.108	0.523 ± 0.122	0.64	0.03 (>)	0.017 (>)
Max. Diameter (mm)	0.578 ± 0.070	0.621 ± 0.089	0.669 ± 0.063	0.050 (<)	^**‡**^0.0001 (<)	0.0054 (<)
Min. Diameter (mm)	0.485 ± 0.072	0.509 ± 0.082	0.563 ± 0.058	0.26	0.0003 (<)	0.0007 (<)
Roundness^+^	0.872 ± 0.070	0.845 ± 0.082	0.886 ± 0.039	0.14	0.79	0.0417 (<)

^+^Roundness = defined by ImageJ – represents how closely region conforms to a circle.

^*^If significant, a greater than or less than sign is presented to dictate which was greater, following the notation given in the group headings. For example, the FAZ area was bigger in the MDR group compared to the healthy control group (see first row and column #6 indicating the p-values for the Healthy vs. MDR).

^**‡**^Indicates a P-value of less than 0.0001.

### Retinal Thickness

Thickness data for each region and layer was tested amongst the three study groups using ANOVA. Due to the volume of data, only significant results in the ETDRS regions from the ANOVA are presented. Kruskal-Wallis test was used to determine inter group significance (Table 2). Thickness data is reported in μm.

**Table 2:** Statistically Significant Thickness Data via ANOVA. P-values are presented from the Kruskal-Wallis test between individual groups. *If significant, a greater than or less than sign is presented to dictate which was greater, following the notation given in the group headings.

Region	Healthy vs. DM*	Healthy vs. MDR*	DM vs. MDR*
RNFL			
I1	0.015 (>)	0.66	0.0015 (<)
I2	0.17	0.28	0.0026 (<)
T2	0.97	0.0081 (<)	0.0006 (<)
GCL + IPL			
S1	0.0051 (>)	0.026 (>)	0.91
I1	0.0016 (>)	0.11	0.21
T1	0.12	0.94	0.03 (<)
T2	0.53	0.0044 (<)	0.0005 (<)
INL			
N2	0.016 (>)	0.048 (>)	0.96
OPL			
T2	0.0025 (<)	0.0004 (<)	0.20
ONL + MZ			
S2	0.0002 (>)	0.0038 (>)	0.95
N2	^**‡**^0.0001 (>)	^**‡**^0.0001 (>)	0.46
I2	0.0022 (>)	0.0081 (>)	0.61
T2	0.0037 (>)	0.033 (>)	0.77
IS			
S2	0.0005 (>)	0.0022 (>)	0.35
I2	0.0003 (>)	0.0055 (>)	0.56
ELZ + OS			
S1	0.0098 (<)	0.81	0.01 (>)
I1	0.075	0.68	0.018 (>)
S2	^**‡**^0.0001 (<)	0.062	0.0021 (>)
N2	0.029 (<)	0.36	0.0008 (>)
I2	^**‡**^0.0001 (<)	0.18	0.0003 (>)
T2	0.0046 (<)	0.77	0.0015 (>)
Inner Retina			
C	0.011 (>)	0.0066 (>)	0.53
S1	0.0011 (>)	0.023 (>)	0.81
I1	0.0006 (>)	0.17	0.054
N2	0.0012 (>)	0.037 (>)	0.52
I2	0.071	0.78	0.014 (<)
T2	0.59	0.0017 (<)	0.0001 (<)
Outer Retina			
C	0.0064 (>)	0.0085 (>)	0.60
I1	0.012 (>)	0.23	0.38
S2	0.0060 (>)	0.019 (>)	0.83
N2	0.0002 (>)	0.0010 (>)	0.91
I2	0.0091 (>)	0.032 (>)	0.98

^**‡**^Indicates a P-value of less than 0.0001.

### Hemodynamic Parameters

**Figure 2:**
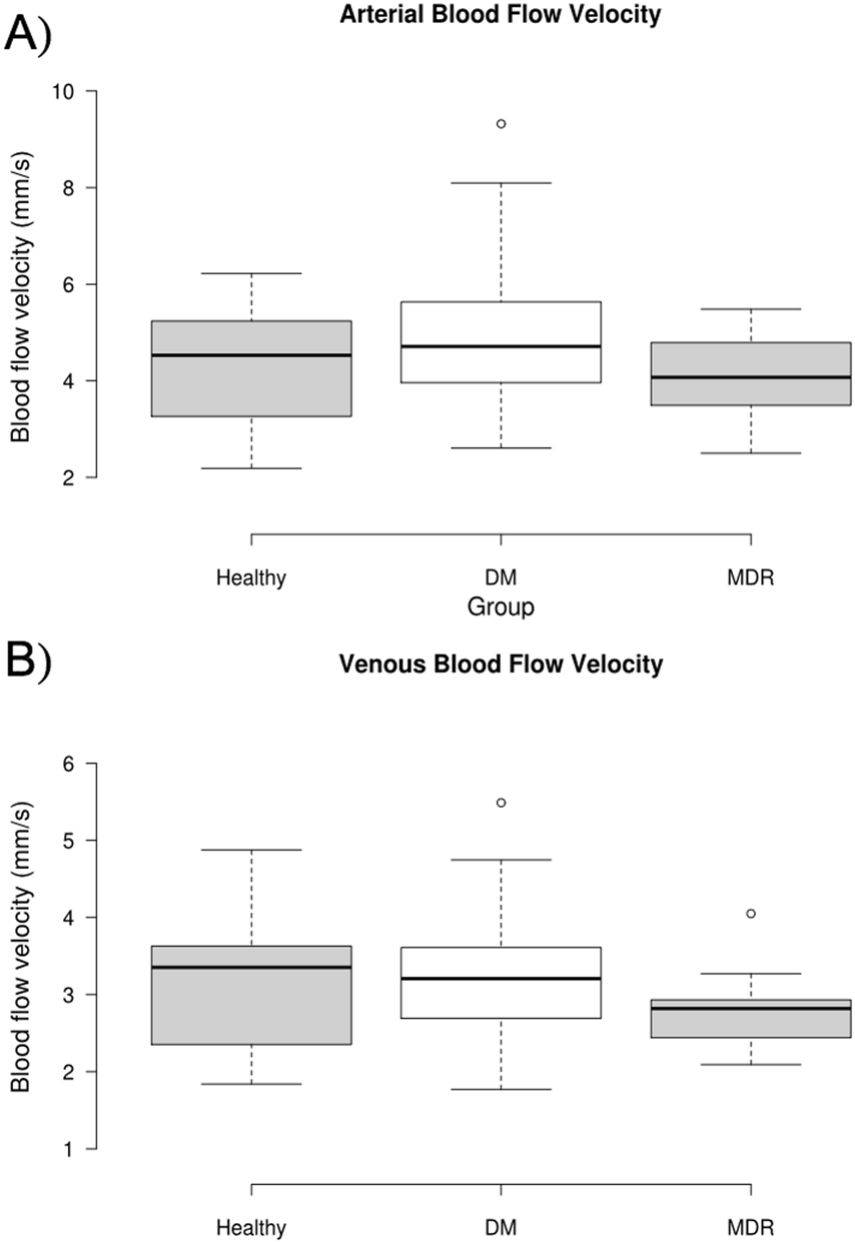
Box plot trends for blood flow velocity in arteries and veins. (**A**) *Arterial Blood Flow Velocity*. The middle 50% of the data groups are as follows: Healthy (3.462–5.550), DM (3.833–5.520), and MDR (3.660–5.472). There was one high outlier. (**B**) *Venous Blood Flow Velocity*. The middle 50% of the data groups are as follows: Healthy (2.443–4.013), DM (2.569–3.581), and MDR (2.432–3.291). There was one high outlier in the DM group and one in the MDR group, which are marked by circles. Outliers were defined as outside of ±3 SD of the mean.

Using the RFI, various hemodynamic parameters could be deduced from the motion of the RBC’s in the retinal vessels. First and foremost, depending on the sign of the velocity, we could determine the identity of the vessel – whether it was an artery or a vein. Also, using the location values for the vessel allowed for the determination of the region that the vessel occupied, whether it be superior or inferior. In this way, we could look at the hemodynamic properties of various regions in the macula and different vessels. A summary of this data is represented in Table 3 (first three columns), where all values are presented in mm/s. ANOVA only showed significance in the veins and superior vein regions. The Kruskal-Wallis results are also displayed in Table 3 (last three columns). Figure 2 shows the box plot trends for the blood flow velocity in arteries and veins.

**Table 3:** Blood flow velocity data for all study groups (first three columns, units: mm/s) and Kruskal-Wallis Test results for blood flow velocity data (last three columns).

Descriptor	Healthy	DM	MDR	Healthy vs. DM*	Healthy vs. MDR*	DM vs. MDR*
Arteries	4.58 ± 1.23	4.70 ± 1.29	4.64 ± 1.31	0.94	0.94	0.64
Veins	3.27 ± 0.900	3.16 ± 0.759	2.88 ± 0.670	0.57	0.016 (>)	0.027 (>)
Superior Arteries	4.55 ± 1.22	4.67 ± 1.39	4.58 ± 1.16	0.99	0.94	0.92
Superior Veins	3.23 ± 0.894	3.18 ± 0.855	2.83 ± 0.856	0.34	0.052	0.016 (>)
Inferior Arteries	4.58 ± 1.40	4.57 ± 1.37	4.51 ± 1.51	0.94	0.62	0.59
Inferior Veins	3.22 ± 1.04	3.10 ± 0.853	2.86 ± 0.679	0.84	0.34	0.30

*If significant, a greater than or less than sign is presented to dictate which was greater, following the notation given in the group headings.

Relationships between FAZ Area and hemodynamic and structural parameters of the macular region. For this portion of the analysis, simple linear regressions were used to deduce whether a relationship existed or not. Each retinal layer and region were tested versus the FAZ area for each study subject, as well as both arterial and venous blood flow velocity in both the superior and inferior regions.

**Figure 3:**
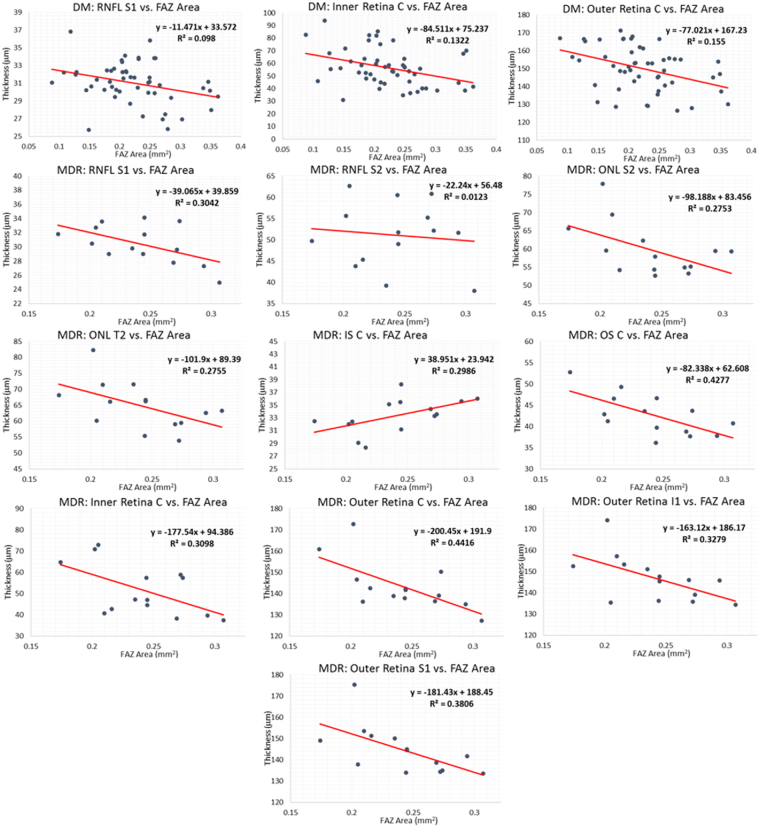
Retinal layer thickness data vs. the FAZ area in the DM and MDR group. Each graph is labeled with the region and layer to which it corresponds. The red line represents the regression line, of which the equation is displayed in the graph region. In all cases, the “y” represents thickness in microns and the “x” represents FAZ area in mm^2^. The R^2^ is the coefficient of determination, which compares the ratio of the average difference of the data point and the line of best fit with that of the data point and the average “y” value. A negative slope corresponds to layer thinning as FAZ area increases, while a positive slope corresponds to layer thickening as FAZ area increases.

The retinal layer thickness data is presented in Fig. 3, with only those with a significant relationship being shown.

The analyses related to the relationships between FAZ area and hemodynamic and structural parameters of the macular region revealed moderate correlation coefficients (R^2^ > 30%) for the central (C), superior (S1) and inferior (I1) regions of the ETDRS grid.

## Discussion

Due to the volume and wide variety of data, many comparisons between study groups were made. These comparisons allow us to understand better the physiological and morphological changes that arise in conditions such as diabetes, as well as in mild diabetic retinopathy.

There were significant variations in the morphology of the FAZ between the three study groups. The FAZ area was not significantly different between healthy controls and DM patients, while the MDR group had a significantly higher FAZ area in comparison to the healthy and DM groups. This trend was the same regarding the FAZ perimeter and the maximum ferret diameter, as expected with the higher value in the area. These results suggest that there is significant enlargement of the FAZ in MDR patients, while there is no significant expansion in DM patients in comparison to healthy controls. Therefore, it may be that the extension of the FAZ is an implication of diabetic retinopathy onset and progression, rather than simply of diabetes mellitus.

There was a consistent significant thickness difference between DM and MDR groups, with DM having a thinner layer in the RNFL and GCL + IPL. In both T2 regions of these layers, the thickness in the MDR group was greater than that of the healthy group. This result may demonstrate that there is a smaller thickness in RNFL and GCL + IPL in patients with diabetes mellitus who do not have DR, while those with mild DR may experience inflammation in these layers.

The INL and OPL have exact opposite trends. The INL (OPL) was significantly thicker (thinner) in the healthy group than in both DM and MDR. In both layers, there was no significant difference between the DM and MDR groups. The OPL thickening observed in the subjects with diabetes mellitus may be associated with the swelling of Müller cells; which have a critical role in facilitating the relationship between neurons and retinal vessels. The ONL + MZ and IS both matched the INL trend, with significantly thicker layers in the healthy group in comparison to both the DM and MDR groups, with no significant difference between DM and MDR.

The ELZ + OS layer showed a consistent significant higher thickness in the DM group in comparison to the MDR and healthy groups. However, it did not show a significant difference in any region between the healthy and MDR groups. Lastly, the inner and outer retina showed consistent results. In most areas, there was a significant higher thickness in the healthy group in comparison to the DM and MDR groups. In the inner retina I2 and T2 regions, there was a significant difference between DM and MDR with MDR showing a greater thickness. However, the outer retina showed no significant difference in any area between DM and MDR.

Considering the number of significant results per layer, the layers and retinal segments showing the most statistically significant differences between groups were ELZ + OS and, the inner and outer retina. This may suggest that these layers and retinal segments might be the most useful for diagnostic comparisons to track the disease onset and progression, as we can demonstrate that there is indeed a thickness difference between the groups.

In comparing the number of significant results per ETDRS’s regions and rings, there were 40 significant results in the 2^nd^ ETDRS ring (6 mm) to only 13 in the 1^st^ ETDRS ring (3 mm). This trend may also suggest that the outer ring may give more meaningful results, or perhaps there is just more variation in thicknesses in the ETDRS regions comprised of this ring.

Regarding the hemodynamic trends, as in previous studies, our results indicated a higher arterial velocity in patients with diabetes mellitus without any evidence of DR pathology in comparison to the healthy group^49–51^. However, a higher speed was not observed in the venous compartments as compared to the healthy group. In previous studies, devices used were different from the RFI not only in the method used to measure the retinal blood flow velocities but also the location and type of vessels studied were different. In this study, a more reliable custom-built algorithm than the RFI’s built-in software available at the time the study was conducted was used to calculate the blood flow velocities^52^. Blood flow velocity measurements were also slower in the MDR group in comparison with the DM group. As anticipated, in all study groups, venous velocity was slower than that in the arteries. Although less clear, the higher blood flow velocities observed in the DM group might indicate that earlier stages of loss of the capillary network and FAZ expansion over time would result in the increased total flow without clinically seen morphological changes.

Also, there were only significant differences present in the venous blood flow velocities. Both the overall and superior venous flows showed significant differences in the MDR group in comparison to the DM group (lower in MDR). Also, the superior venous flow showed a significant decrease in MDR group compared to the healthy group. This result may suggest that there is a slower venous blood flow, specifically in the superior region, in patients with mild DR. This trend could be attributed to vasoconstriction and perifoveal capillary loss which is demonstrated by a higher enlargement of the FAZ in this group. There were no significant differences in arterial blood flow, as well as no differences in the inferior region. Therefore, venous blood flow may be a better indicator of disease onset and progression. These blood flow results are expected as diabetes often leads to a variety of microvascular complications, leading to a decrease in blood flow to many regions of the body. This poor circulation may affect the eye, which can be manifested as diabetic retinopathy. In more severe cases, we may see an even larger decrease in blood flow when compared to both healthy and diabetic with no retinopathy groups.

The results for the relationship between the FAZ area and the structural parameters only showed significance in the thicknesses of a few layers and regions in the DM and MDR groups. There was no significance in the blood flow velocity in any of the groups, and there was no significance in the thicknesses in the healthy control group. Capillaries near the FAZ transport a relatively small blood flow. In our study, capillaries bordering the avascular zone showed slowed flow consistent with this trend in all groups. In DR, there are patterns of capillary loss in both the macula and especially in the mid-periphery as the disease progresses. A previous study reported a significant negative correlation between capillary blood flow velocity and the size of the FAZ in patients with Type 2 diabetes without edema using FA^53^. However, this study included Type 2 DM patients with different severity of retinopathy (i.e. mild, moderate and severe with nonproliferative and proliferative retinopathy) in the same group for the analyses. The lack of significance between the FAZ and the blood flow velocity in our study may be influenced by no significant alterations in the local capillary network structure at earlier stages when loss of the capillary network and FAZ expansion may be occurring slowly. Results that confirm such assumption have been obtained, but they are not part of this publication. However, this result might also suggest that the blood flow changes in early DR are a primary issue and not secondary to underlying microvascular network changes (i.e. FAZ enlargement).

There was a significantly smaller value of thickness in MDR patients in a variety of layers as the FAZ area was larger: RNFL (S1, I1), ONL + MZ (S2, T2), ELZ + OS (C), Inner Retina (C), and Outer Retina (C, S1, I1). One layer and region showed a higher thickness as a function of FAZ area, which was the IS in the central region (IS C). The DM group showed significantly smaller values of thickness as a function of the FAZ area in 3 areas: RNFL S1, Inner Retina C, and Outer Retina C. The FAZ area was larger in the MDR group, and a more asymmetric FAZ was observed in the DM group. This result may suggest a potential anisotropy in the mechanical properties of the diabetic retina with no retinopathy that may trigger the elongation in a preferred horizontal or vertical direction resulting in thinning or thickening of intraretinal layers in the inner and outer segment as a result of autoregulation or early damage in the outer blood retinal barrier in diabetes.

In comparing the measurements for the DM and MDR groups in ETDRS regions with significant thickness differences, the slopes of the regression models (which represent how many micrometers the thickness is altered by per 1 mm^2^ area increase) were used. It is worth noticing that the slope measures obtained for the MDR group in the ETDRS regions were more than double that the slope values in the DM group, which may suggest that there is a sharper decrease in thickness as the FAZ area increases in MDR patients. Extensively modeling these parameters may offer more insight into this, allowing for a more in depth knowledge of these relationships and how they interact.

The relatively large standard deviations of some parameters shown in Tables 1 and 3 may be related to the individual characteristics and healthy status of the study subjects. The FAZ area in the superficial plexus was found variable (mean ± SD = 0.266 ± 0.097) in a study population of healthy subjects^22^. A more recent study has also reported the same variability in a larger cohort of healthy subjects (mean ± SD = 0.32 ± 0.11 mm^2^)^54^. The individual variability also point to the need to introduce a quantitative measure not prone to individual differences in FAZ morphology. Also, although the literature using the RFI for BF data analysis documents the macular circulation variability per individuals that suffer from diabetic eye complications and other diseases affecting the eye health^55,56^, the variability in our BFV parameters is justified by the fact that capillary dropout was identified in some study subjects with diabetes mellitus. As a potential explanation, the same blood volume flows through a smaller overall diameter (because of the diameter of the missing capillaries that have dropped out) which makes velocities larger in regions closer to the capillary droupout areas (e.g. see outliers in Fig. 2). Therefore, the measurement variability are mostly reflecting the real conditions of the subjects. Also, our results support the outcomes of prior studies on the relationship of FAZ area and central retinal thickness, and we also found that FAZ area enlargement may be more associated with the outer retinal structure in the central region of the ETDRS grid at the early stage of retinopathy^20,21,57,58^.

The present study has some limitations that need to be mentioned. The sample size was small, and only subjects with Type 2 diabetes were included. Therefore, the degree to which our results can be generalized to individuals with Type 1 diabetes is uncertain. Particularly, retinal structure and function may be affected by factors such as hyperlipidemia, older age, and hypertension in Type 2 diabetes. Consequently, the early vascular disease in patients with Type 2 diabetes may have a different origin than in Type 1 diabetes. Also, although the linear relationship assumption was mainly based on previous studies^20,21,57,58^, our correlation results might be influenced by the small sample size and differences in the number of samples per group. Therefore, the correlations rather show a trend and are not meant to be used either for diagnosis or therapy. Our regression results rather shed light on potential correlations between retinal microstructure and FAZ area. Moreover, regarding the R^2^ values, we note these values show that the given retinal structural changes are only in part responsible for the FAZ area changes (e.g. the outer retinal thinning in MDR eyes is in 44% responsible for the FAZ enlargement in the central region, i.e. there are other factors in this region justified by the remaining 56%). This is no surprising, it is a multifactorial change; and probably a multivariate regression analysis with a larger sample size would render higher correlations, and even might reveal a different law for such a relationship. However, investigations using further studies are warranted and may show that such hypotheses hold through in DR and even in other retinal diseases. Last but not least, proper understanding of individual differences in relation to the normal characteristics of the retinal structure as well as the potential variations by individual traits, such as sex/gender and race/ethnicity are important when making clinical evaluations to detect abnormal conditions.Therefore, because previous studies^59–68^ have reported differences in foveal structure in individuals with different race, ethnicity and gender, further studies are necessary to determine whether the results of the present study are influenced by race, ethnicity and gender.

## Conclusion

This study identified a variety of parameters that may play a role in DR and the development of the disease in patients suffering from diabetes. These parameters included FAZ characteristics, including area and diameter, intraretinal layer thicknesses as defined in a variety of ETDRS regions, and blood flow analysis through the retinal microvasculature.

In this study, these parameters were compared between the groups, noting statistically significant differences. There was a higher FAZ area and diameter in MDR patients in comparison to healthy and DM patients, suggesting that FAZ enlargement may be a marking feature of the disease onset and progression. Several retinal thickness differences were also identified in a variety of layers and regions between the groups, allowing for the understanding of how these layers may change throughout the early onset and progression of DR. Blood flow analysis also showed that there is a significantly smaller venous blood flow velocity in MDR patients, which highlights the microvascular changes in the retina.

Lastly, significant regression relationships between retinal thickness and the FAZ area were defined. About ten such relationships were found in the MDR group, three were found in the DM group, and none were found in the healthy control group. This result may suggest that the FAZ area may be a better indicator of retinal thickness alteration in MDR patients, while it may have little to no effect in healthy controls patients as expected. In comparing the ETDRS regions with significant differences that the DM and MDR groups shared in common, there was a more than two-fold reduced thickness per unit area in the MDR group for these ETDRS regions. Also, a significant difference in roundness was observed between DM and MDR groups supporting the development of asymmetrical FAZ expansion with worsening DR^35^. Interestingly, the FAZ area was larger in the MDR group but more asymmetric in the DM group. This result suggests a potential anisotropy in the mechanical properties of the diabetic retina with no retinopathy that may trigger the FAZ elongation in a preferred direction resulting in either thinning or thickening of intraretinal layers in the inner and outer segments of the retina as a result of autoregulation or early damage in the outer blood retinal barrier in diabetes.

Despite numerous studies investigating the FAZ and its relation to the retinal microstructure, there have been no studies that quantify the irregularity of the FAZ and correlate this metric to both the retinal microcirculation and microstructure as a function of disease stage^33,69,70^. Our study introduced a multimodal optical imaging approach that facilitated metrics to characterize hemodynamic disturbances such as altered blood flow and, quantify both the irregularity of the FAZ and remodeling of the retinal microstructure. Gaining an in depth understanding of these differences and relationship trends is important to understand DR and how it progresses, as well as ensuring the disease is diagnosed accurately and early to promote better clinical outcomes. With further longitudinal studies, these preliminary findings may be enhanced and built upon to allow us to detect better and quantify the changes associated with the onset and progression of DR.

## Methods

### Study Population

The study was approved by the Institutional Review Board (University of Miami, Miami, FL, USA). The research adhered to the tenets outlined in the Declaration of Helsinki and written informed consent was obtained from each subject. In this prospective study, enrollment was offered to diabetic patients referred to the comprehensive ophthalmology clinic that had diabetic retinopathy up to ETDRS level 35 and without macular edema, as well as diabetic patients with no retinopathy^71^.

Patients with proliferative disease, clinically significant macular edema (CSME), and anatomic abnormalities that might confound the evaluation of macular architecture, such as glaucoma, vitreoretinal traction, and epiretinal membranes were excluded. Patients with medical conditions that might affect visual function, receiving treatments with medications that might affect retinal thickness (e.g., chloroquine or niacin containing anti-cholesterol agents), recent cataract surgery, previous vitrectomy, or unstable blood sugars were excluded.

After informed consent most patients enrolled in our study had Type 2 DM. The routine ophthalmic examination was carried out with dilated fundoscopy and patients were divided into two groups based on the absence (DM group) and mild DR (MDR group). Any eyes with more severe DR were excluded from the study. Advanced imaging was carried out by Spectralis SD-OCT (Heidelberg Engineering GmbH, Heidelberg, Germany) and Retinal Function Imager (RFI, Optical Imaging, Ltd., Rehovot, Israel). In this investigation, study subjects (age-matched) were selected from a 5-years longitudinal cohort based on the quality of imaging data required to perform all analyses. Table 4 shows the demographics of the study population.

**Table 4:** Study Participant Demographics.

Descriptor	Healthy	DM	MDR
Number of patients (Male/Female)	29 (7/22)	45 (11/34)	25 (7/18)
Number of Eyes (OD/OS)	54 (27/27)	82 (42/40)	40 (23/17)
Mean age ± SD, yrs	52.4 ± 5.12	53.5 ± 7.81	52.8 ± 7.10

### Optical Coherence Tomography Imaging and Quantitative Analysis

OCT is one of the most commonly utilized diagnostic tools in the practice of ophthalmology, providing a non-invasive and high-resolution approach to serve as a diagnostic technique for a variety of retinal pathologies^36^. OCT allows for the imaging of the retinal layers, with subtle boundaries able to be detected and studied individually^1^. This utility has been expanded into the realm of research, facilitating researcher’s ability to track the course of the disease and better assess the *in-vivo* conditions of their study patients.

Study subjects were scanned using a Spectral Domain OCT (Spectralis SD-OCT, Heidelberg Engineering, Germany) unit using the IR + OCT protocol, with a 30° IR scan angle and 30° and 25° (8.5 × 7.1 mm) OCT pattern. This imaging modality operates under a diode with a wavelength of 870 mm, providing an axial resolution of 4μm and acquisition speeds of up to 40,000 A-scans per second. Image contrast was enhanced using Tru-Track^TM^ active eye tracking technology, which averages five aligned images to reduce noise. The produced images were 768 × 61 × 496 pixels, with a transversal resolution of 11.11μm/pixel and an axial resolution of 3.87 μm/pixel. This data consisted of a confocal scanning laser ophthalmology (cSLO) image and 61 B-scans of 496 × 768 pixels^72^.

**Figure 4:**
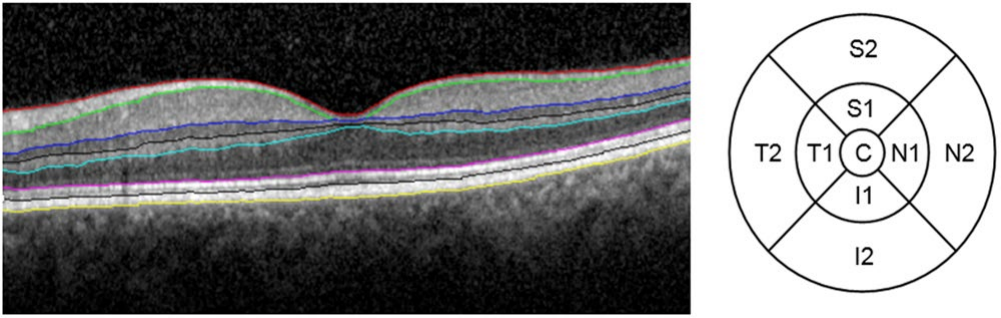
Left, segmentation of a macular OCT B-scan. The layers from top to bottom are the RNFL, GCL + IPL, OPL, ONL + MZ, ELZ + OS, IDZ + RPE/BC. Right, the nine ETDRS regions with their locations, highlighted in a right eye. The concentric rings for inner (1) and outer (2) used were 3 mm and 6 mm in diameter, respectively. (Abbreviations: RNFL, retinal nerve fiber layer; GCL + IPL, ganglion cell layer and inner plexiform layer complex; INL, inner nuclear layer; ONL, outer nuclear layer; MZ, myoid zone; ELZ, ellipsoid zone; OS, outer segment; OPL, outer plexiform layer; RPE/BC, retinal pigment epithelium/Bruch’s complex. Also, ETDRS Grid for OD eye with nine regions denoted. C1 = central retina, N1 = inner nasal, S1 = inner superior, T1 = inner temporal, I1 = inner inferior, N2 = outer).

The retinal layer thickness analysis was accomplished by using our custom-built OCTRIMA3D segmentation software. This algorithm has shown to have an accuracy that competed with other commonly used automated software^40,41,70^. A total of seven layers were extracted in all scans and an average thickness was calculated in the nine ETDRS regions for all single and composite retinal layers (RNFL: retinal nerve fiber layer; GCL + IPL: ganglion cell layer and inner plexiform layer complex; INL: inner nuclear layer; OPL: outer plexiform layer; ONL + MZ: outer nuclear layer and myoid zone; ELZ + OS, ellipsoid zone and outer segment and; RPE/BC: retinal pigment epithelium/Bruch’s complex; Inner retina (IR = RNFL + GCL + IPL), Outer retina (OR = OPL + INL + ONL + MZ) and Total retina (TR). These layers are shown in Fig. 4. The processing time of OCTRIMA3D was 20 seconds for the automatic segmentation. Manual correction was needed in some cases and it took approximately 5–15 minutes per volume data depending on the corrections needed per B-scan.

### Imaging and Hemodynamic Analysis by the Retinal Function Imager

Visualizing and quantizing the function and morphology of the retinal capillary network can be an extremely useful tool in the early diagnosis of a variety of pathologies, including DR^42,43^. Many techniques have been developed over time to acquire higher quality images to aid in this early detection, with the current gold standard being Fluorescein Angiography (FA)^44^. However, this modality is limited in the fact that it is used repeatedly throughout treatment, which results in a higher risk of complications related to the fluorescein dye used in the procedure^46^. OCT angiography (OCTA) is a recently advanced technique that is revolutionizing the visualization of the morphology and circulation features of the eye in three-dimensions. However, it was not commercially available in 2010 when our longitudinal study was initiated^73^.

The RFI utilizes a noninvasive method of imaging through acquiring images at a wavelength that is absorbed strongly by the hemoglobin that is found in the RBCs^47,48^. A green (“red-free”) light with a spectrum of 548 ± 75 mm is used for illumination, with a typical 17.5 milliseconds between each flash. Throughout this period, eight images are captured with a resolution of 1024 × 1024 pixels. The area corresponding to this is 4.3 × 4.3 mm or 7.2 × 7.2 mm dependent on the choice of field of view, 20° or 35°, respectively.

The hemodynamic analysis was done using our custom-built software^52^. A main advantage of the software is that the fovea detection occurs using the SLO component of the OCT data collected which is then overlaid onto the RFI image coordinates, where the lowest value of the thickness is detected as the foveal center point. Then, both major and minor vessels in the RFI are tracked by combining the key frame and non-invasive capillary perfusion map (nCPM) image, which greatly enhances contrast. The vessel boundary is detected using a short-path graph search on an edge-weighted graph^52^. Lastly, blood flow velocities were calculated by tracking the blood cells along the centerline of the vessels in the ratio video.

### Fovea and FAZ Morphology Analysis

The foveal morphology analysis was performed on the nCPM composite images, which were generated from the individual scans taken with the RFI^52^. These capillary maps were then used to gather information regarding the FAZ, which can be identified in the image as the central area where no vasculature is present. An active contouring model was used to identify and outline the FAZ^74^. The active contouring program is run by constant user supervision; therefore each run was reviewed ensuring that it accurately represented the FAZ^57^. If it appeared to be inaccurate, the simulation was rerun with different parameters, specifically with a different region of interest drawn to capture the area better. If it was still not representative of the actual FAZ, the image was excluded from the analysis due to poor quality. This process was done blindly, in that the group of the patient was unknown.

Following identification of the FAZ using active contouring, the images were analyzed using ImageJ (National Institutes of Health, Bethesda, MD)^75^. This software allows for the easy acquisition of data regarding the region of interest, including area, circumference, perimeter, and the maximum/minimum Feret diameter^76^. The Feret diameter is based on directionality – it is tested at each angle in the image, and the maximum and minimum are calculated based on this process.

### Statistical Analysis

A one-way ANOVA test was used to determine if there was a difference present in any group, and then a post hoc Kruskal-Wallis test was used to discern these individual group differences^77,78^. For the relationship between FAZ area and the other parameters, a linear regression correlation test was used. A p-value of 0.05 was used in all cases to define significance. The SD method was used to detect outliers in the data (mean ± 3 SD). Correlations were classified into ranges of importance: not statistically significant; significant, but weak, |R| < 0.32 (R^2^ < 10%) (p < 0.05); modest, |R| from 0.32 to 0.55 (R^2^ from 10%-30%); and moderate, |R| > 0.55 (R^2^ > 30%).

### Ethical approval

All procedures performed in studies involving human participants were in accordance with the ethical standards of the institutional and/or national research committee and with the 1964 Helsinki declaration and its later amendments or comparable ethical standards.
